# Eosinophil-to-monocyte ratio is a potential biomarker in the prediction of functional outcome among patients with acute ischemic stroke

**DOI:** 10.1186/s12868-021-00610-x

**Published:** 2021-02-05

**Authors:** Shuhong Yu, Yi Luo, Tan Zhang, Chenrong Huang, Yu Fu, Qiang Zhang, Fangyue Zeng, Hao Huang, Chunyuan Zhang, Zhiliang Guo

**Affiliations:** 1 Department of Encephalopathy, Suzhou Hospital of Integrated Traditional Chinese and Western Medicine, Suzhou, 215101 China; 2 Department of Neurology and Suzhou Clinical Research Center of Neurological Disease, The Second Affiliated Hospital of Soochow University, No. 1055, Sanxiang Road, Suzhou, 215004 Jiangsu China

**Keywords:** Eosinophil-to-monocyte ratio (EMR), Ischemic stroke, Outcome, Biomarker

## Abstract

**Background:**

It has been shown that eosinophils are decreased and monocytes are elevated in patients with acute ischemic stroke (AIS), but the impact of eosinophil-to-monocyte ratio (EMR) on clinical outcomes among AIS patients remains unclear. We aimed to determine the relationship between EMR on admission and 3-month poor functional outcome in AIS patients.

**Methods:**

A total of 521 consecutive patients admitted to our hospital within 24 h after onset of AIS were prospectively enrolled and categorized in terms of quartiles of EMR on admission between August 2016 and September 2018. The endpoint was the poor outcome defined as modified Rankin Scale score of 3 to 6 at month 3 after admission.

**Results:**

As EMR decreased, the risk of poor outcome increased (*p* < 0.001). Logistic regression analysis revealed that EMR was independently associated with poor outcome after adjusting potential confounders (odds ratio, 0.09; 95% CI 0.03–0.34; *p* = 0.0003), which is consistent with the result of EMR (quartile) as a categorical variable (odds ratio, 0.23; 95% CI 0.10–0.52; *p*_trend_ < 0.0001). A non-linear relationship was detected between EMR and poor outcome, whose point was 0.28. Subgroup analyses further confirmed these associations. The addition of EMR to conventional risk factors improved the predictive power for poor outcome (net reclassification improvement: 2.61%, *p* = 0.382; integrated discrimination improvement: 2.41%, *p* < 0.001).

**Conclusions:**

EMR on admission was independently correlated with poor outcome in AIS patients, suggesting that EMR may be a potential prognostic biomarker for AIS.

## Background

Inflammatory and immunological responses play pivotal roles in the pathogenesis of acute ischemic stroke (AIS) which is still a main challenge to public health [1–3]. Leukocytes including monocytes, neutrophils, and lymphocytes are indispensable inflammatory cells and are associated with endothelial dysfunction, thrombosis, blood–brain barrier disruption and tissue damage in AIS [1–4]. Moreover, eosinophils have also been shown to be inflammatory cells and were able to regulate the inflammatory responses by facilitating the resolution of inflammation [5]. Recent studies have discovered that stroke triggers an acute decrease in circulating eosinophil counts and an increase in circulating monocytes [6]. Furthermore, post-stroke low circulating eosinophil count was inversely associated with stroke severity and risk of mortality, and high peripheral blood monocyte level was associated with high risk of poor outcome after stroke [7, 8]. Since the single inflammatory cell is unable to summarize the overall systematic inflammation, new indexes by combining different subtypes of the leukocyte are needed [3, 9]. Given the deleterious effects of classical monocytes and the possible neuroprotective effect of eosinophils in stroke [5, 10–12], eosinophil-to-monocyte ratio (EMR), a novel biomarker reflecting the integrated application value of eosinophils and monocytes, is needed to identify patients at high risk of poor prognosis.

In this study, we aimed to determine the relationship between EMR on admission and 3-month poor functional outcome, and to explore the predictive value of EMR in the poor functional outcome in patients with AIS.

## Methods

### Study population

Consecutive patients with ischemic stroke admitted to the department of encephalopathy at our hospital from August 2016 to September 2018 were prospectively recruited. The inclusion criteria for enrollment were as follows: (1) diagnosis of AIS according to the World Health Organization criteria based on patient history, clinical data, and neuroimaging results (computed tomography or magnetic resonance imaging) [13]; (2) time from onset of stroke to hospitalization was < 24 h; and (3) the patient or their relatives provided informed consent. Study exclusion criteria were: (1) asthma, eosinophilic esophagitis, hypereosinophilic syndrome, evidence of active infection, chronic inflammatory, autoimmune diseases, steroid therapy, cancer, blood system diseases, previous stroke with partial recovery, severe hepatic or renal dysfunction (90 patients); (2) unavailable complete blood cell count or medical records (48 patients); (3) lost to follow-up (n = 29). At last, 521 consecutive ischemic stroke patients were included in the current study. (flowchart of participants selection: Additional file 1: Fig. S1). The study protocol was approved by the institutional Human Research Ethics Committees of Suzhou Integrated Traditional and Western Medicine Hospital, and written informed consent was obtained from all participants or their authorized relatives.

### Clinical protocol and laboratory tests

Medical history including potential stroke risk factors, clinical examination, blood tests, 12-lead electrocardiogram and treatment administration were performed at admission. Stroke severity was assessed by a certified neurologist using the National Institutes of Health Stroke Scale (NIHSS) at admission. The etiologic subtypes of ischemic stroke were classified according to the TOAST (Trial of ORG 10172 in Acute Stroke Treatment) criteria [14]. Given the relatively small number of participants in the other determined etiology and unknown subtypes, we combined these two groups into one group (other etiology/unknown).

In all patients, peripheral venous blood samples were obtained for the measurement of leucocytes, the value of EMR and measuring serum lipid levels. EMR was calculated by dividing eosinophil count by monocyte count. The follow-up data were achieved by telephone. The endpoint was the poor outcome at month 3 after admission with a modified Rankin Scale (mRS) score of 3–6.

### Statistical analysis

The total procedure of statistical analysis was divided into five steps. First, baseline characteristics of study participants were presented according to the quartiles of EMR. The one-way ANOVA (normal distribution), Kruskal–Wallis H (skewed distribution) test and chi-square test (categorical variables) were used to determine any significant differences between groups according to the quartiles of EMR. Second, we used a univariate regression model to evaluate the associations between EMR and 3-month poor outcome in patients with AIS. For multivariate analysis, we first included age and sex (model 1) and then included variables in the final models if they were significantly associated with poor outcome (*p* < 0.10) or changed the estimates of EMR on poor outcome by more than 10% (model 2; age, sex, history of hyperlipidemia, history of previous stroke, history of atrial fibrillation, ischemic stroke subtypes, triglyceride, NIHSS score at baseline, premorbid mRS score and proton pump inhibitors treatment). Additional file 1: Tables S2–S5 show the associations of each confounder with the outcomes of interest [15, 16]. Third, we used generalized additive models (GAM) to identify the non-linear relationships because EMR was a continuous variable. If a non-linear relationship was observed, a two-piecewise linear regression model was used to calculate the threshold effect of the EMR on poor outcome in terms of the smoothing plot. When the ratio between EMR and poor outcome appeared obvious in a smoothed curve, the recursive method automatically calculates the inflection point, where the maximum model likelihood will be used [15]. Fourth, we conducted subgroup analyses to assess the robustness of association between low EMR and poor outcome of AIS by using of stratified linear regression models. The modifications and interactions between EMR and subgroup variables on the poor outcome were tested by likelihood ration tests [16]. Fifth, receiver operating characteristic (ROC) curves were used to test the overall discriminative ability of the EMR, eosinophils and monocytes for poor outcome. The differences in discriminative ability were tested using the DeLong method [17]. Moreover, we constructed a conventional model (only including conventional risk factors: age, sex, history of hyperlipidemia, history of previous stroke, history of atrial fibrillation, ischemic stroke subtypes, triglyceride and NIHSS score at baseline) and a new model (including conventional risk factors and EMR) by logistic regression model. To assess the improvement in risk prediction for poor prognosis of ischemic stroke by adding EMR to conventional risk factors, we calculated net reclassification improvement (NRI) and integrated discrimination improvement (IDI) through comparing these 2 models. All analyses were performed with the statistical software package R (http://www.R-project.org, The R Foundation) and EmpowerStats (http://www.empowerstats.com, X&Y Solutions, Inc., Boston, MA). A two-sided *p* value < 0.05 was considered to be statistically significant [15].

## Results

### Baseline characteristics of patients

Most of the baseline characteristics were balanced between patients included and patients excluded (Additional file 1: Table S1). A total of five hundred and twenty-one patients with ischemic stroke were included in the current study and the average age was 69 years. Main baseline characteristics of study participants according to quartiles of EMR are presented in Table 1. The participants with lower EMR values tended to be older, female and to have subtype of cardioembolism, and had higher NIHSS score, history of hyperlipidemia, higher high-density lipoprotein cholesterol levels, and lower levels of triglyceride. These patients also were more likely to have poor outcome (Table 1).

**Table 1:** Baseline characteristics of study participants according to quartiles of eosinophil-to-monocyte ratio (EMR)

Characteristics	Q1	Q2	Q3	Q4	*p* value
No. of patients	125	135	130	131	
Age, years, mean (SD)	72.54 (13.50)	70.09 (12.48)	67.58 (12.64)	67.59 (13.96)	0.006
Female, (%)	64 (51.20%)	69 (51.11%)	50 (38.46%)	49 (37.40%)	0.026
Hypertension, (%)	113 (90.40%)	109 (80.74%)	113 (86.92%)	110 (83.97%)	0.150
Diabetes, %	34 (27.20%)	31 (22.96%)	34 (26.15%)	28 (21.37%)	0.671
Hyperlipidemia, %	36 (28.80%)	53 (39.26%)	55 (42.31%)	65 (49.62%)	0.008
Previous stroke, %	26 (20.80%)	28 (20.74%)	21 (16.15%)	21 (16.03%)	0.594
Coronary heart disease, %	16 (12.80%)	10 (7.41%)	14 (10.77%)	11 (8.40%)	0.461
Atrial fibrillation, %	26 (20.80%)	25 (18.52%)	19 (14.62%)	16 (12.21%)	0.245
Current cigarette smoking, %	14 (11.20%)	8 (5.93%)	19 (14.62%)	13 (9.92%)	0.138
NIHSS, median (IQR)	4 (2–8)	4 (2–6)	3 (1–6)	2 (1–6)	0.005
Premorbid mRS score, median (IQR)	0 (0–0)	0 (0–0)	0 (0–0)	0 (0–0)	0.293
Stroke subtypes					0.006
Large artery atherosclerosis	40 (32.00%)	61 (45.19%)	48 (36.92%)	39 (29.77%)	
Cardioembolic stroke	21 (16.80%)	22 (16.30%)	17 (13.08%)	17 (12.98%)	
Small artery disease	59 (47.20%)	52 (38.52%)	64 (49.23%)	75 (57.25%)	
Other etiology/unknown	5 (4.00%)	0 (0.00%)	1 (0.77%)	0 (0.00%)	
IV rtPA, %	7 (5.60%)	13 (9.63%)	9 (6.92%)	16 (12.21%)	0.237
Proton pump inhibitors, %	56 (44.80%)	54 (40.00%)	59 (45.38%)	44 (33.59%)	0.186
Triglyceride, mmol/L, median (IQR)	1.20 (0.89–1.60)	1.38 (1.00–1.82)	1.40 (0.94–1.92)	1.52 (1.06–2.38)	0.005
Total cholesterol, mmol/L, mean (SD)	4.77 (1.06)	4.99 (1.18)	4.92 (1.37)	5.00 (1.04)	0.364
High-density lipoprotein cholesterol, mmol/L, mean (SD)	1.36 (0.35)	1.34 (0.39)	1.28 (0.37)	1.24 (0.37)	0.035
Low-density lipoprotein cholesterol, mmol/L, mean (SD)	2.37 (0.70)	2.52 (0.74)	2.48 (0.82)	2.53 (0.70)	0.290
EMR, median (IQR)	0.04 (0.01–0.05)	0.12 (0.10–0.15)	0.26 (0.21–0.30)	0.49 (0.40–0.76)	< 0.001
Poor outcome	65 (52.00%)	53 (39.26%)	34 (26.15%)	29 (22.14%)	< 0.001

*EMR* eosinophil-to-monocyte ratio, *IQR* interquartile range, *IV rtPA* intravenous recombinant tissue plasminogen activator, *NIHSS* National Institutes of Health Stroke Scale

### Univariate and multivariate analysis

The results of univariate analysis showed that age, female, history of previous stroke, history of coronary heart disease, history of atrial fibrillation, subtype of cardioembolism, NIHSS score at baseline, premorbid mRS score and proton pump inhibitors treatment were positively correlated with poor outcome, whereas history of hyperlipidemia, triglyceride, subtype of small-artery occlusion and EMR were negatively associated with poor outcome (Table 2).

**Table 2:** The results of univariate analysis

Characteristics	Poor outcome		*p* value
	No	Yes	
Age, years, mean (SD)	66.90 ± 12.68	74.17 ± 13.09	< 0.001
Female, (%)	140 (41.18%)	92 (50.83%)	0.035
Hypertension, (%)	285 (83.82%)	160 (88.40%)	0.159
Diabetes, (%)	77 (22.65%)	50 (27.62%)	0.208
Hyperlipidemia, (%)	147 (43.24%)	62 (34.25%)	0.046
Previous stroke, (%)	49 (14.41%)	47 (25.97%)	0.001
Coronary heart disease, (%)	20 (5.88%)	31 (17.13%)	< 0.001
Atrial fibrillation, (%)	29 (8.53%)	57 (31.49%)	< 0.001
Current cigarette smoking, (%)	33 (9.71%)	21 (11.60%)	0.499
NIHSS, median (IQR)	2 (1–3)	7.00 (4–11)	< 0.001
Premorbid mRS score, median (IQR)	0 (0–0)	0 (0–0)	0.065
Stroke subtypes			< 0.001
Large artery atherosclerosis	108 (31.76%)	80 (44.20%)	
Cardioembolic stroke	25 (7.35%)	52 (28.73%)	
Small artery disease	201 (59.12%)	49 (27.07%)	
Other etiology/unknown	6 (1.76%)	0 (0.00%)	
IV rtPA, (%)	29 (8.53%)	16 (8.84%)	0.904
Proton pump inhibitors	106 (31.18%)	107 (59.12%)	< 0.001
Triglyceride, mmol/L, median (IQR)	1.46 (1.02–2.03)	1.21 (0.94–1.67)	0.001
Total cholesterol, mmol/L, mean (SD)	4.91 ± 1.45	4.94 ± 1.15	0.765
High-density lipoprotein cholesterol, mmol/L, mean (SD)	1.29 ± 0.37	1.33 ± 0.38	0.289
Low-density lipoprotein cholesterol, mmol/L, mean (SD)	2.46 ± 0.74	2.50 ± 0.74	0.577
EMR, median (IQR)	0.22 (0.10–0.39)	0.12 (0.05–0.23)	< 0.001

*EMR* eosinophil-to-monocyte ratio, *IQR* interquartile range, *IV rtPA* intravenous recombinant tissue plasminogen activator, *NIHSS* National Institutes of Health Stroke Scale

Table 3 summarizes the results of multivariate logistic regression model. The EMR as a continuous variable was independently associated with a smaller risk of poor outcome with an adjusted odds ratio (OR) of 0.23 [95% confidence interval (CI) 0.09–0.56] after adjustment for age and sex (model 1) and 0.09 (0.03–0.34) after adjustment for all potential covariates (model 2). For the purpose of sensitivity analysis, we converted the EMR into categorical variable by quartile and calculated *p* for trend, the OR (95% CI) of poor outcome for those in the highest quartile of EMR were 0.23 (0.10–0.52) compared with patients in the lowest quartile of EMR. The *p* for trend was < 0.0001.

**Table 3:** Relationship between eosinophil-to-monocyte ratio (EMR) and poor outcome among patients with acute ischemic stroke in different models

Variable	Non-adjusted model		Model 1		Model 2	
	OR (95% CI)	*P*	OR (95% CI)	*P*	OR (95% CI)	*P*
EMR	0.19 (0.08, 0.46)	0.0002	0.23 (0.09, 0.56)	0.0011	0.09 (0.03, 0.34)	0.0003
EMR (quartile)						
Q1	1.0		1.0		1.0	
Q2	0.60 (0.36, 0.98)	0.0398	0.64 (0.38, 1.07)	0.0869	0.56 (0.29, 1.10)	0.0903
Q3	0.33 (0.19, 0.55)	< 0.0001	0.38 (0.22, 0.66)	0.0005	0.27 (0.13, 0.59)	0.0010
Q4	0.26 (0.15, 0.45)	< 0.0001	0.30 (0.17, 0.52)	< 0.0001	0.23 (0.10, 0.52)	0.0005
*P* for trend	< 0.0001		< 0.0001		< 0.0001	

Non-adjusted model: we did not adjust other covariates

Model 1: we adjusted age and sex

Model 2: we adjusted age, sex, history of hyperlipidemia, history of previous stroke, history of atrial fibrillation, ischemic stroke subtypes, triglyceride, NIHSS score at baseline, premorbid mRS score and proton pump inhibitors treatment

*EMR* eosinophil-to-monocyte ratio, *OR* odds ratio

### The analyses of non-linear relationship

**Fig. 1:**
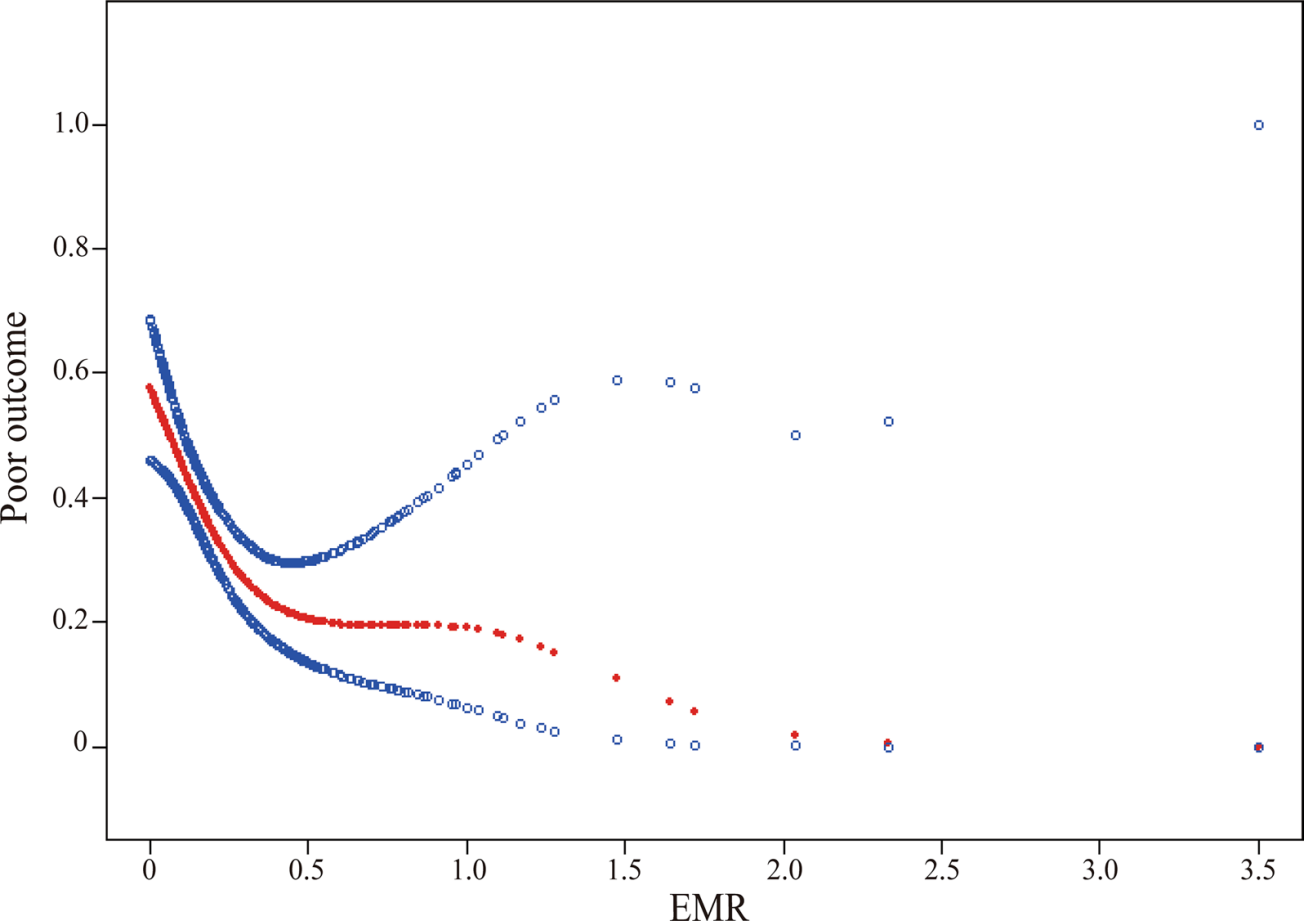
The non-linear relationship between eosinophil-to-monocyte ratio (EMR) and poor outcome of ischemic stroke. A nonlinear relationship between them was detected after adjusting for age, sex, history of hyperlipidemia, history of previous stroke, history of atrial fibrillation, ischemic stroke subtypes, triglyceride, NIHSS score at baseline, premorbid mRS score and proton pump inhibitors treatment

In the present study, we analyzed the non-linear relationship between EMR and poor outcome (Fig. 1). The result of smooth curve showed that the relationship between EMR and poor outcome was non-linear after adjustment for all potential covariates (*p* = 0.0008). We compared linear regression model (fitting the relationship between EMR and poor outcome by a linear) and two-piecewise linear regression model (fitting the relationship between EMR and poor outcome by a curve) (Table 4). The *p* for log likelihood ratio test is 0.013 which is less than 0.05. This result indicates that the two-piecewise linear regression model should be used to fit the relationship between EMR and poor outcome. By using a two-piecewise linear regression model, we calculated the inflection point was 0.28. On the left of inflection point, the effect size was 0.00 (95% CI 0.00–0.06, *p* = 0.0002). However, on the right side of the inflection point, we did not observe a significant association between EMR and poor outcome (0.42, 95% CI 0.09–1.94, *p* = 0.2656; Table 4).

**Table 4:** The results of two-piecewise linear regression model

Outcome:	Effect size OR (95% CI)	*p* value
Inflection point of EMR	0.28	
< 0.28	0.00 (0.00, 0.06)	0.0002
≥ 0.28	0.42 (0.09, 1.94)	0.2656
*p* for log likelihood ratio test	0.013	

Effect: EMR; cause: poor outcome; adjusted: age, sex, history of hyperlipidemia, history of previous stroke, history of atrial fibrillation, ischemic stroke subtypes, triglyceride and NIHSS score at baseline

*EMR* eosinophil-to-monocyte ratio, *OR* odds ratio

### The results of subgroup analyses

As is shown in Table 5, the test of interactions was significant for NIHSS (*p* for interaction = 0.0023), while the tests of interactions were not statistically significant for age, sex, history of hypertension, history of diabetes mellitus, history of hyperlipidemia, history of previous stroke, history of coronary heart disease, history of atrial fibrillation, current cigarette smoking, ischemic stroke subtypes, receiving treatment with intravenous recombinant tissue plasminogen activator and proton pump inhibitors treatment (*p* values for interaction were larger than 0.05). The effect sizes of EMR on poor outcome showed significant differences in different NIHSS score. EMR was negatively associated with poor outcome in participants with minor stroke (NIHSS score < 4) (0.00, 95% CI 0.00–0.03, *p* = 0.0006). However, the effect sizes of EMR on poor outcome was statistically non-significant in participants with NIHSS score ≥ 4 (*p* = 0.0789; *p* for interaction = 0.0023 in the model 2).

**Table 5:** Subgroup analyses on the association between eosinophil-to-monocyte ratio (EMR) and poor outcome of ischemic stroke

Subgroup	No. of participants	OR (95% CI)	*p* value	*p* for interaction
Age, years				0.2716
< 65	179	0.37 (0.04, 3.57)	0.3900	
≥ 65	342	0.08 (0.02, 0.35)	0.0008	
Sex				0.1422
Male	289	0.08 (0.02, 0.43)	0.0033	
Female	232	0.05 (0.01, 0.28)	0.0010	
Hypertension				0.4943
No	76	35.05 (0.00, Inf)	0.9999	
Yes	445	0.08 (0.02, 0.35)	0.0006	
Diabetes				0.4333
No	394	0.11 (0.03, 0.40)	0.0008	
Yes	127	0.00 (0.00, 0.04)	0.0027	
Hyperlipidemia				0.8288
No	312	0.13 (0.02, 0.71)	0.0187	
Yes	209	0.05 (0.00, 0.62)	0.0202	
Previous stroke				0.1904
No	425	0.06 (0.01, 0.26)	0.0002	
Yes	96	0.23 (0.01, 8.31)	0.4233	
Coronary heart disease				0.5110
No	470	0.08 (0.02, 0.32)	0.0004	
Yes	51	Inf^a^ (0.00, Inf^a^)	0.9997	
Atrial fibrillation				0.5915
No	435	0.08 (0.02, 0.34)	0.0008	
Yes	86	0.10 (0.01, 2.02)	0.1344	
Current cigarette smoking				0.7324
No	467	0.09 (0.02, 0.35)	0.0004	
Yes	54	0.00 (0.00, Inf^a^)	0.9992	
NIHSS				0.0023
< 4	295	0.00 (0.00, 0.03)	0.0006	
≥ 4	226	0.37 (0.12, 1.12)	0.0789	
Stroke subtypes				0.9674
Large artery atherosclerosis	188	0.13 (0.02, 0.81)	0.02856	
Cardioembolic stroke	77	0.13 (0.01, 2.35)	0.1659	
Small artery disease	250	0.06 (0.00, 0.73)	0.0280	
Other etiology/unknown	6	Inf^a^		
IV rtPA				0.7945
No	476	0.09 (0.02, 0.34)	0.0005	
Yes	45	0.00 (0.00, Inf^a^)	0.9993	
Proton pump inhibitors				0.3518
No	308	0.03 (0.00, 0.26)	0.0016	
Yes	213	0.19 (0.04, 0.91)	0.0381	

In the multivariate models, confounding factors, such as age, sex, history of hyperlipidemia, history of previous stroke, history of atrial fibrillation, ischemic stroke subtypes, triglyceride, NIHSS score at baseline, premorbid mRS score and proton pump inhibitors treatment were included unless the variable was used as a stratification variable

*EMR* eosinophil-to-monocyte ratio, *IQR* interquartile range, *IV rtPA* intravenous recombinant tissue plasminogen activator, *NIHSS* National Institutes of Health Stroke Scale, *OR* odds ratio

^a^The model failed because of the small sample size

### Incremental prognostic value of EMR in patients with ischemic stroke

ROC curves comparing the discrimination performance of EMR, eosinophil and monocyte count on poor outcome are shown in Additional file 1: Fig. S2. The area under the curve (AUC) of EMR (AUC: 0.6532; 95% CI 0.6032–0.7031) for poor outcome is greater than those of eosinophils (AUC: 0.6257; 95% CI 0.5746–0.6769; *p* = 0.0007) and monocytes (AUC: 0.5831; 95% CI 0.5317–0.6344; *p* = 0.0452). In addition, we examined whether adding serum EMR to the conventional risk factors improved the risk prediction of clinical outcomes after AIS. And we found adding EMR to conventional risk factors significantly improved predictive power for poor outcome (NRI: 2.61%, *p* = 0.382; IDI: 2.41%, *p* < 0.001).

## Discussion

Previous studies have shown that stroke triggers an acute decrease in circulating eosinophil counts and an increase in circulating monocytes [6]. Furthermore, post-stroke low circulating eosinophil count was inversely associated with stroke severity and risk of mortality, and high peripheral blood monocyte level was associated with high risk of poor outcome after stroke [7, 8]. These studies, however, did not control for stroke severity and have other issues such as its retrospective nature or the small sample size that limit interpretation of the findings. In addition, all of these studies are mainly focused on a single subpopulation of leukocytes, which may not provide a comprehensive study for the eosinophils and monocytes [7, 8]. Given the deleterious effects of classical monocytes and the possible neuroprotective effect of eosinophils in stroke [5, 10–12], EMR, a novel biomarker reflecting the integrated application value of eosinophils and monocytes, is needed to identify patients at high risk of poor prognosis [9]. Like other study in different diseases [9], EMR is used to identify patients at high risk of poor outcome in stroke, and we found the lower EMR on admission was associated with higher risk of 3-month poor functional outcome in patients with AIS, and also proved EMR was of certain value in predicting poor outcome in patients with AIS.

Firstly, we demonstrated that there was a saturating effect on the linear relationship between EMR and poor outcome in the present study. The inflection point we calculated by the recursive algorithm was 0.28. The result means the negative linear association between EMR and poor outcome is only present in participants with relatively low EMR level. For those with relatively high EMR level, this linear relationship cannot be found. There is no current study to explore the non-linear relationships between EMR and poor outcome, but the previous investigations might give us some clues. A recent study revealed that the adjusted odds ratios reflecting the effect sizes of eosinophils on cerebral infarct volume in the Q4 (the reference group), Q3, Q2 and Q1 group were 1.00, 2.416, 4.988 and 50.791, respectively. Similarly, on the effect sizes of eosinophils on poor outcome, the odds ratios in 4 quartiles of eosinophils (from high to low) were 1.00, 2.747, 4.804 and 30.2991, respectively [7]. Moreover, on the effect sizes of monocytes on novel plaque formation, the odds ratios in 4 quartiles of monocytes (from low to high) were 1.00, 1.17, 1.12 and 1.85, respectively [18]. This kind of non-equidistant changes in effect size suggested that there may be a nonlinear relationship between EMR and poor outcome. Given that GAM can handle non-parametric smoothing and has obvious advantages in dealing with non-linear relations, the use of GAM will help us to better discover the real relationships between EMR and poor outcome [15]. Therefore, we use the GAM to clarify their nonlinear relationship in the present study and a nonlinear relationship between EMR and poor outcome was detected after adjusting for potential covariates.

Secondly, we eliminated the confounding effects of the severity of AIS by adjusting baseline NIHSS in the multivariable models and performing the collinearity screening and subgroup analyses. Given that the percentage of eosinophils was negatively correlated with infarct volume and eosinopenia had the potential to predict the severity of AIS [7, 19, 20], we performed the collinearity screening and found there was not collinearity between EMR and NIHSS (Additional file 1: Table S2). Moreover, we found that the significant association of EMR with poor outcome was independent of the baseline NIHSS, especially in participants with minor stroke (NIHSS score < 4; *p* for interaction = 0.0023 in the model 2) in the multivariate analysis and further subgroup analyses. These results suggested that EMR had additional prognostic value when baseline NIHSS was considered, and we should apply EMR for the prediction of poor outcome in participants with minor stroke. The reason why the effect sizes of EMR on poor outcome showed significant differences in different NIHSS score remains unclear. We hypothesized that the complication of patients with severe stroke and higher NIHSS itself might cause some disruptions to the relationship between EMR and poor outcome. Further studies are needed to investigate this hypothesis.

Thirdly, we also explored whether EMR had greater and additional prognostic value for poor outcome by using of various statistical methods. First, the AUC of EMR for poor outcome is greater than that of eosinophil or monocyte count, suggesting that EMR is superior to only the eosinophil count or monocyte count for distinguishing the occurrence of poor outcome. The reasons for such a superiority of EMR may relate to what the EMR represents. The EMR reflects the balance between eosinophil and monocyte levels, which may be comprehensively summarize the overall systematic inflammation conditions [9]. Second, given that the guide advises the visual representation of the relationship between predicted and observed (not the specific *p* value) is the best way to evaluate calibration [21], our results suggested EMR could significantly improve the predictive power for poor outcome beyond established traditional risk factors (NRI: 2.61%; IDI: 2.41%).Therefore, we hypothesized that serum EMR might be useful in risk stratification of poor outcome among patients with ischemic stroke and could assist the selection of high-risk patients in future clinical practice. If patients have low EMR levels at admission, they may be at high risk of poor outcome and should receive aggressive monitoring and therapeutic interventions.

The mechanisms underlying these observations are not well established, but they seem to be related to the roles of eosinophils and monocytes in ischemic insult. Eosinophils can secrete over 35 cytokines, growth factors and chemokines, including IL-4, IL-13 and vascular endothelial growth factor (VEGF). IL-4 and IL-13 are capable of inducing the activation of the M2 phenotype microglia, which possess neuroprotective properties by facilitating the resolution of inflammation. And VEGF might be neuroprotective by the modulation of angiogenesis [5, 10, 11]. The phenotypes of monocytes include CD14^high^CD16^−^ (classical monocytes), CD14^dim^CD16^+^ and CD14^high^CD16^+^ [12]. It is worth noting that the classical monocytes account for nearly 90%, which have deleterious effects after stroke. Nevertheless, the other two phenotypes contribute about 10% of monocytes, which play a beneficial role in patients with stroke [12]. Therefore, the comprehensive effects of peripheral blood monocytes reflect the roles of classical monocytes during the study of monocytes as a whole. Given the deleterious effects of classical monocytes and the possible neuroprotective effect of eosinophils [5, 10–12], our study found the lower EMR on admission was associated with higher risk of 3-month poor functional outcome in patients with AIS.

The main strength of our study is that the clinical information and blood samples of all patients were collected in a prospective fashion with a relatively large sample size.

In addition, we provided a comprehensive study for the eosinophils and monocytes by combining the two into a novel biomarker (EMR). Nonetheless, our current findings also have some limitations. First, the majority of acute critical patients were transferred to higher level hospitals due to the grass-roots hospitals nature of ours, which might result in the baseline NIHSS scores being relatively low with NIHSS median 3 (1–6) and existing deviations of the enrolled patients. Moreover, the baseline information of endovascular treatment which can impact stroke outcome was not been presented in the present study due to lack of this treatment in our hospital [22]. Thus, the findings might not generalize to other populations, particularly those with high NIHSS scores or experiencing endovascular treatment. Second, the predictive value of EMR for poor outcome is relatively low compared to other study (area under the curve: 0.6532 in stroke vs. 0.789 or 0.752 in patients with ST-elevation myocardial infarction) [9]. The reasons for the low predictive value may relate to the complicated roles of eosinophils and monocytes in ischemic insult, which have proinflammatory or anti-inflammatory properties [5, 10–12]. For example, we did not categorize the monocytes subpopulations which include CD14^high^CD16^−^, CD14^dim^CD16^+^ and CD14^high^CD16^+^ phenotypes [12]. The other reason may relate to the timing chose for the EMR analysis, because it is unknown whether EMR is a dynamic variable as is the case with NLR [3]. Further studies are needed to investigate whether EMR is a dynamic variable and whether the subsequent EMR provide a greater predictive value for poor outcome than the baseline EMR. Third, we neither explored the mechanisms by which eosinophils and monocytes affected the neurovascular unit damage nor investigated what factors regulated the changes of eosinophils and monocytes after ischemic strokes in animal studies. These are going to be the focus of our next work, especially exploring the role of eosinophils in AIS and its mechanism.

## Conclusions

In summary, we found that lower EMR on admission was associated with higher risk of 3-month poor functional outcome, indicating that EMR may be a potential prognostic biomarker for AIS. Further studies from other samples of patients with AIS are needed to validate our results.

## Supplementary Information

**Additional file 1: Table S1.** Demographic and clinical characteristics of included and excluded patients. **Table S2.** The collinearity screening of baseline characteristics. **Table S3.** Associations of covariates with poor outcome (N = 521). **Table S4.** The adjusting roles of potential confounders on the estimates of EMR on poor outcome. **Table S5.** The selected covariates. **Figure S1.** Flow chart of patient cohort. **Figure S2.** Prognostic value of eosinophil-to-monocyte ratio (EMR) in patients with ischemic stroke.

## Data Availability

The datasets used and/or analyzed during the current study are available from the corresponding author on reasonable request.

## Abbreviations

AIS: Acute ischemic stroke
AUC: Area under the curve
CI: Confidence interval
EMR: Eosinophil-to-monocyte ratio
GAM: Generalized additive models
IDI: Integrated discrimination improvement
IQR: Interquartile range
IV rtPA: Intravenous recombinant tissue plasminogen activator
mRS: Modified Rankin Scale
NIHSS: National Institutes of Health Stroke Scale
NRI: Net reclassification improvement
OR: Odds ratio
ROC: Receiver operating characteristic
TOAST: Trial of ORG 10172 in Acute Stroke Treatment
