# Supplementary material for: Eosinophil-to-monocyte ratio is a potential biomarker in the prediction of functional outcome among patients with acute ischemic stroke
Source: BMC Neurosci. 2021 Feb 5;22:8. doi: 10.1186/s12868-021-00610-x (PMC7863468; doi:10.1186/s12868-021-00610-x)
Supplement: Supplementary file 1 — Additional file 1: Table S1. Demographic and clinical characteristics of included and excluded patients. Table S2. The collinearity screening of baseline characteristics. Table S3. Associations of covariates with poor outcome (N = 521). Table S4. The adjusting roles of potential confounders on the estimates of EMR on poor outcome. Table S5. The selected covariates. Figure S1. Flow chart of patient cohort. Figure S2. Prognostic value of eosinophil-to-monocyte ratio (EMR) in patients with ischemic stroke. [file 12868_2021_610_MOESM1_ESM.doc]

**Additional file 1**

**Eosinophil-to-monocyte ratio is a potential biomarker in the prediction of functional outcome among patients with acute ischemic stroke**

**Table S1.** Demographic and clinical characteristics of included and excluded patients

| Characteristics | Patients included | Patients excluded | *p* |
| --- | --- | --- | --- |
| No. of patients | 521 | 167 |  |
| Age, years, mean (SD) | 69.42 (13.27) | 68.97 (12.38) | 0.696 |
| Female, (%) | 232 (44.53%) | 70 (41.92%) | 0.554 |
| Hypertension, (%) | 445 (85.41%) | 137 (82.04%) | 0.293 |
| Diabetes, % | 127 (24.38%) | 31 (18.56%) | 0.120 |
| Hyperlipidemia, % | 209 (40.12%) | 74 (44.31%) | 0.338 |
| Previous stroke, % | 96 (18.43%) | 39 (23.35%) | 0.163 |
| Coronary heart disease, % | 51 (9.79%) | 25 (14.97%) | 0.063 |
| Atrial fibrillation, % | 86 (16.51%) | 38 (22.75%) | 0.068 |
| Current cigarette smoking, % | 54 (10.36%) | 20 (11.98%) | 0.559 |
| Baseline NIHSS, median (IQR) | 3 (1-6) | 3 (1-5) | 0.427 |
| Stroke subtypes |  |  | <0.001 |
| Large artery atherosclerosis | 188 (36.08%) | 57 (34.13%) |  |
| Cardioembolic stroke | 77 (14.78%) | 36 (21.56%) |  |
| Small artery disease | 250 (47.98%) | 65 (38.92%) |  |
| Other etiology/unknown | 6 (1.15%) | 9 (5.39%) |  |
| IV rtPA, % | 45 (8.64%) | 16 (9.58%) | 0.709 |

IV rtPA indicates intravenous recombinant tissue plasminogen activator; NIHSS, National Institutes of Health Stroke Scale; SD, standard deviation.

**Table S2.** The collinearity screening of baseline characteristics

| Characteristics | Variance inflation factor |
| --- | --- |
| Age | 1.3 |
| Female | 1.3 |
| Hypertension | 1.1 |
| Diabetes | 1.1 |
| Hyperlipidemia | 1.8 |
| Previous stroke | 1.1 |
| Coronary heart disease | 1.2 |
| Atrial fibrillation | 1.5 |
| Current cigarette smoking | 1.1 |
| NIHSS | 1.2 |
| Premorbid mRS score | 1.5 |
| Stroke subtypes | 1.1 |
| IV rtPA | 1.1 |
| Proton pump inhibitors | 1.2 |
| Triglyceride | 2 |
| Total cholesterol | 4.3 |
| High-density lipoprotein cholesterol | 1.5 |
| Low-density lipoprotein cholesterol | 3.4 |
| EMR | 1.1 |

EMR, eosinophil-to-monocyte ratio; IV rtPA, intravenous recombinant tissue plasminogen activator; NIHSS, National Institutes of Health Stroke Scale.

We think collinearity exists and eliminate these covariables in the final models if their variance inflation factor are greater than or equal to 5.

**Table S3. Associations of covariates with poor outcome (N = 521)**

| Covariates | exp(beta) | 95%CI | *p* value |
| --- | --- | --- | --- |
| Age | 1.0475 | 1.0311-1.0641 | <0.0001 |
| Female | 1.4767 | 1.0275-2.1224 | 0.0352 |
| Hypertension | 1.4703 | 0.8579-2.5200 | 0.1608 |
| Diabetes | 1.3037 | 0.8625-1.9705 | 0.2083 |
| Hyperlipidemia | 0.6840 | 0.4704-0.9948 | 0.0469 |
| Previous stroke | 2.0830 | 1.3289-3.2650 | 0.0014 |
| Coronary heart disease | 3.3067 | 1.8245-5.9930 | 0.0001 |
| Atrial fibrillation | 4.9296 | 3.0105-8.0721 | <0.0001 |
| Current cigarette smoking | 1.2210 | 0.6840-2.1798 | 0.4995 |
| NIHSS | 1.4853 | 1.3795-1.5992 | <0.0001 |
| Premorbid mRS score | 1.6372 | 1.1113-2.4119 | 0.0126 |
| Stroke subtypes |  |  |  |
| Large artery atherosclerosis | Reference | Reference | Reference |
| Cardioembolic stroke | 2.8080 | 1.6075-4.9050 | 0.0003 |
| Small artery disease | 0.3291 | 0.2150-0.5037 | <0.0001 |
| Other etiology/unknown | 0.0000 | 0.0000- Inf ^a^ | 0.9795 |
| IV rtPA | 1.0399 | 0.5489-1.9700 | 0.9044 |
| Proton pump inhibitors | 3.1920 | 2.1943-4.6434 | <0.0001 |
| Triglyceride | 0.8277 | 0.6894-0.9937 | 0.0426 |
| Total cholesterol | 1.0238 | 0.8776-1.1942 | 0.7649 |
| High-density lipoprotein cholesterol | 1.2965 | 0.8023-2.0952 | 0.2889 |
| Low-density lipoprotein cholesterol | 1.0718 | 0.8406-1.3667 | 0.5758 |

EMR, eosinophil-to-monocyte ratio; IV rtPA, intravenous recombinant tissue plasminogen activator; NIHSS, National Institutes of Health Stroke Scale.

^a^ The model failed because of the small sample size.

**Table S4. The adjusting roles of potential confounders on the estimates of EMR on poor outcome**

| +/- covariates | Basic model | Complete model | The selected covariates |
| --- | --- | --- | --- |
| Initial regression coefficient of EMR | -1.6736 | -2.2907 |  |
| Age | -1.4838 ^a^ | -2.1110 | Yes |
| Female | -1.5935 | -2.2384 |  |
| Hypertension | -1.6478 | -2.3016 |  |
| Diabetes | -1.6559 | -2.3342 |  |
| Hyperlipidemia | -1.6197 | -2.2687 |  |
| Previous stroke | -1.6258 | -2.2503 |  |
| Coronary heart disease | -1.6555 | -2.2474 |  |
| Atrial fibrillation | -1.5644 | -2.3066 |  |
| Current cigarette smoking | -1.6892 | -2.2483 |  |
| NIHSS | -2.1036 ^a^ | -1.5697 ^a^ | Yes |
| Premorbid mRS score | -1.6034 | -2.3517 |  |
| Stroke subtypes | -1.6461 | -2.2000 |  |
| IV rtPA | -1.6839 | -2.3413 |  |
| Proton pump inhibitors | -1.5940 | -2.3108 |  |
| Triglyceride | -1.5932 | -2.3446 |  |
| Total cholesterol | -1.6834 | -2.2979 |  |
| High-density lipoprotein cholesterol | -1.6499 | -2.2286 |  |
| Low-density lipoprotein cholesterol | -1.6876 | -2.2641 |  |

^a^ These confounders changed the estimates of EMR on poor outcome by more than 10% when introduce covariates into the basic model or remove covariates from the complete model.

**Table S5.** The selected covariates

| Y | X | The selected covariates（Criterion 1） | The selected covariates（Criterion 2） |
| --- | --- | --- | --- |
| Poor outcome | EMR | Age, NIHSS | Age, sex, history of hyperlipidemia, history of previous stroke, history of atrial fibrillation, ischemic stroke subtypes, triglyceride, NIHSS, premorbid mRS score and proton pump inhibitors |

Criterion 1: These confounders changed the estimates of EMR on poor outcome by more than 10% when introduce covariates into the basic model or remove covariates from the complete model (Table IV)

Criterion 2: These variables were significantly associated with poor outcome (P < 0.10) or changed the estimates of EMR on poor outcome by more than 10% (Table III + Table IV)

**Figure S1.** Flow chart of patient cohort


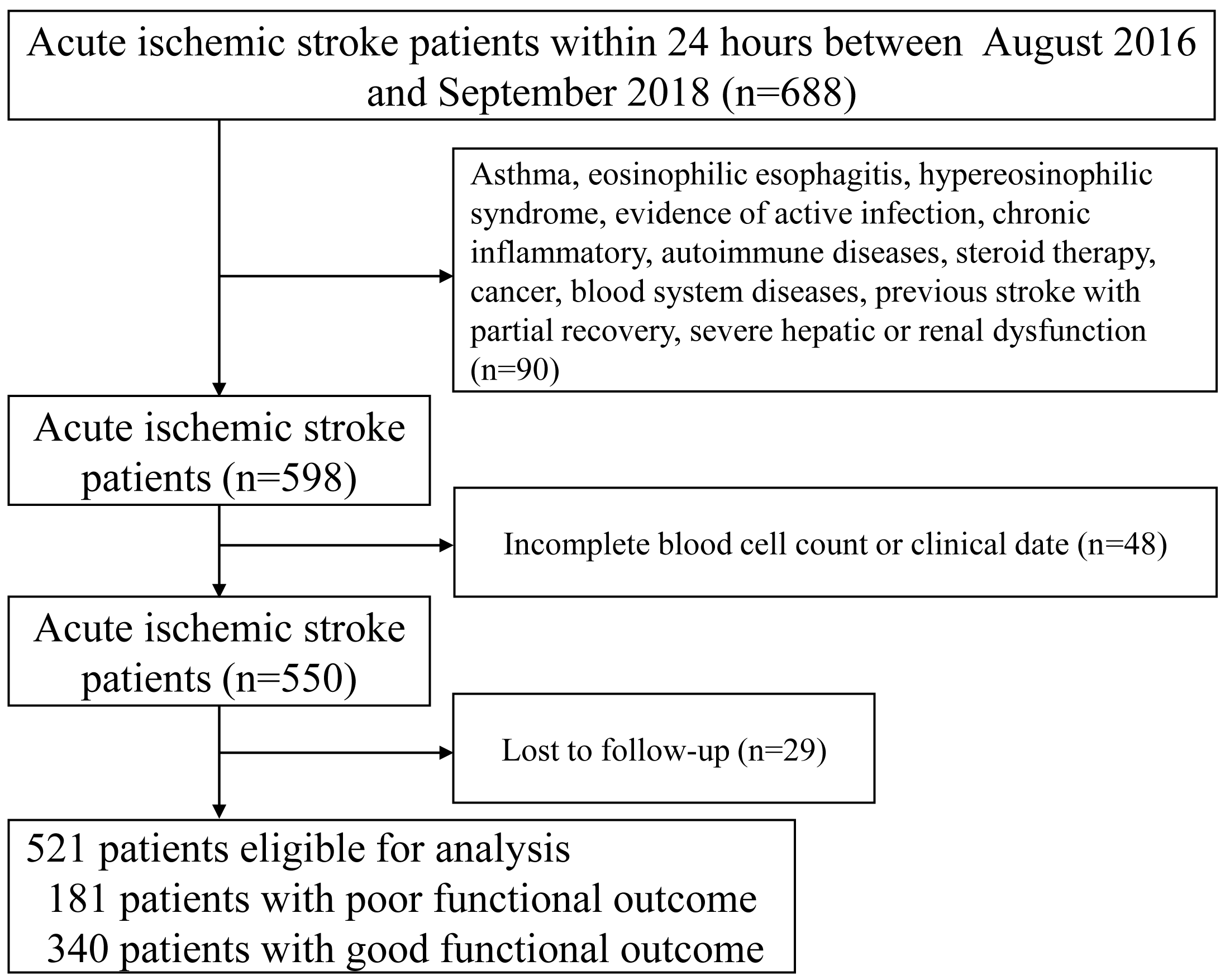


**Figure S2.** Prognostic value of eosinophil-to-monocyte ratio (EMR) in patients with ischemic stroke


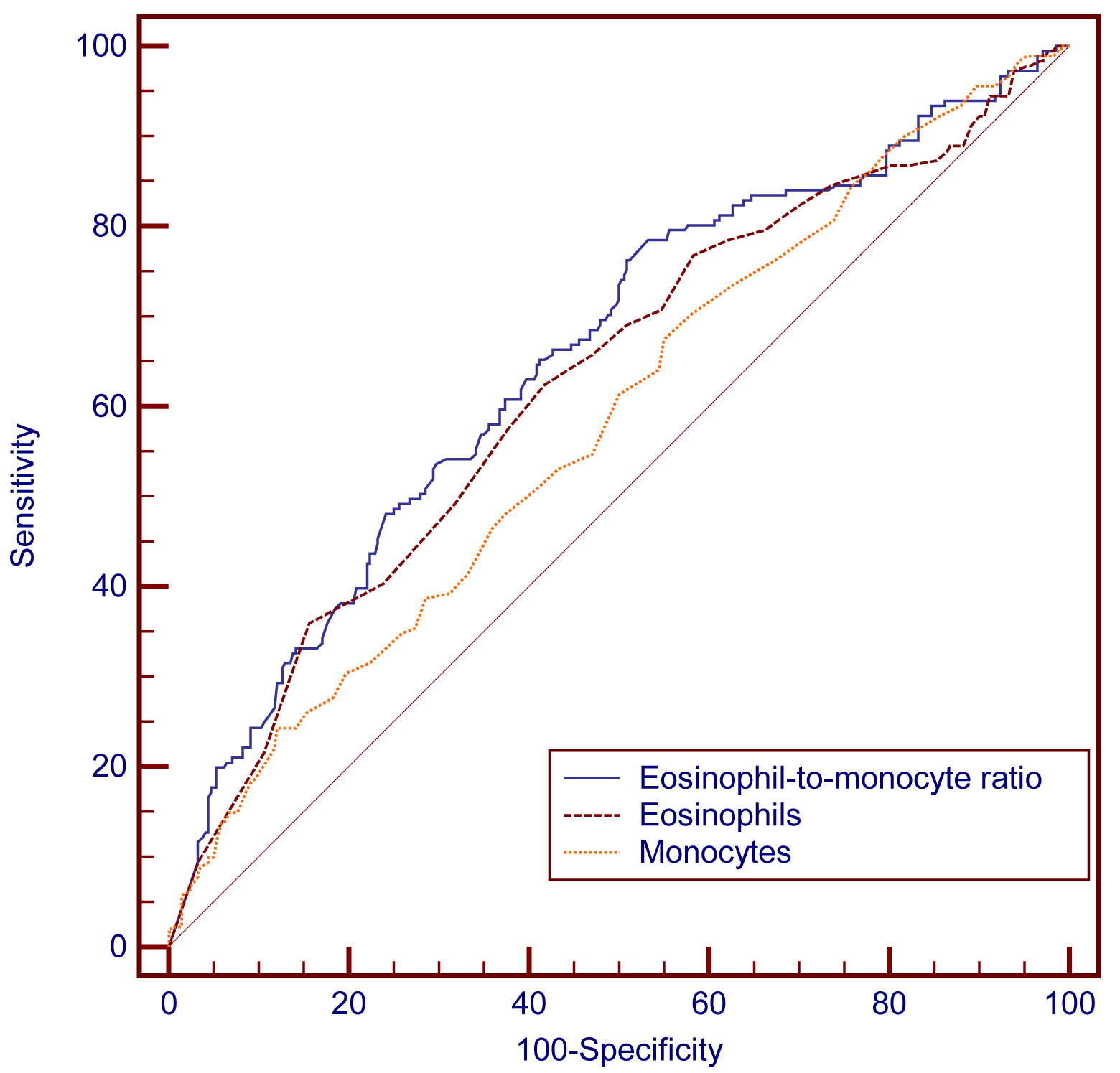


Receiver operator characteristic (ROC) curves comparing the discrimination performance of EMR, eosinophils and monocytes on poor outcome.
